# LncRNA OIP5-AS1 accelerates intervertebral disc degeneration by targeting miR-25-3p

**DOI:** 10.1080/21655979.2021.2007697

**Published:** 2021-12-07

**Authors:** Zhaoping Che, Jie Xueqin, Zongyu Zhang

**Affiliations:** a Department of Operation, The Traditional Chinese Medical Hospital of Lianyungang, Lianyungang, Jiangsu Province, China; b Department of Operation, The Second People’s Hospital of Lianyungang, Lianyungang Jiangsu Province, China; c Department of Orthopaedics, The Traditional Chinese Medical Hospital of Lianyungang, Lianyungang Jiangsu Province, China

**Keywords:** Intervertebral disc degeneration, Nucleus pulposus cells, OIP5-AS1, MiR-25-3p

## Abstract

It is obvious that epigenetic processes influence the evolution of intervertebral disc degeneration (IDD). However, its molecular mechanisms are poorly understood. Long noncoding RNAs (lncRNAs) have been validated to exert vital roles in IDD. Therefore, we tested the hypothesis that OIP5-AS1, a potential regulator of IDD, modulates IDD progression. RT-PCR was utilized to detect levels of OIP5-AS1, miR-25-3p, Collagen II and Aggrecan in IDD tissues and nucleus pulposus cells (NPCs). Immunofluorescence assay measured Collagen II expression. CCK-8, EdU, and flow cytometry estimated the levels of proliferation and apoptosis. Proteins were assessed via Western blot. The binding affinity of OIP5-AS1 with miR-25-3p was investigated by luciferase reporter assay. Enzyme-linked immunosorbent assay (ELISA) analyzed the levels of inflammatory factors. OIP5-AS1 was high expressed in IDD tissues and its expression gradually promoted with the increasing of Pfirrmann scores. The cell morphology of NPCs changed into spindle-shaped, and Collagen II expression was low. After OIP5-AS1 was silenced, cell proliferation was boosted whereas both apoptosis and extracellular matrix (ECM) degradation were restrained. In LPS-activated NPCs, OIP5-AS1 depletion also suppressed inflammation response. Further, miR-25-3p was a target of OIP5-AS1. The effects of OIP5-AS1 silence on proliferation, apoptosis, and ECM degradation were reversed upon miR-25-3p downregulation. Moreover, the inhibitory impact of OIP5-AS1 knockdown on the inflammation of LPS-treated NPCs was rescued with miR-25-3p inference. In general, lncRNA OIP5-AS1 exerted its effects in IDD by targeting miR-25-3p, implying the usage of OIP5-AS1/miR-25-3p as a novel regulatory axis for the molecular targets of IDD therapy.

## Introduction

Intervertebral disc degeneration (IDD) is a type of multi-factorial disease that frequently causing low back pain [1,2]. It is attributed to internal and external factors such as senescence, genetics, autoimmune, as well as mechanical compression [3,4], accompanied with complex etiology [5]. In spinal surgery, some nursing methods are commonly used as assistants. For example, surgical safety nursing, surgical environment care, nursing care of electrosurgical operation. Further, IDD imposes a heavy burden and socioeconomic costs on patients globally [6,7]. It is therefore of great significance to seek for the potential modulatory axis in IDD.

Compelling evidence has illustrated that during the pathogenesis of IDD, long noncoding RNAs (lncRNAs) are pivotal molecules by functioning in nucleus pulposus cells (NPCs) [8,9]. LncRNAs are long RNA transcripts with size over 200 nucleotides (nt) and incapable of encoding proteins [10,11]. However, the amounts of researches studying the role of lncRNAs in IDD remain rare. Among dysregulated lncRNAs, lncRNA OIP5 antisense RNA 1 (OIP5-AS1) is one functional molecule in diverse diseases. For instance, OIP5-AS1 facilitates the apoptosis of oxidized low density lipoprotein (ox-LDL)-treated vascular endothelial cells via GSK-3β by EZH2 recruitment [12]. Depletion of OIP5-AS1 promotes cell viability, hinders apoptosis and restricts LDH release in ox-LDL-induced HUVECs [13]. Qu et al identify novel IDD‑associated lncRNAs have found that the lncRNA OIP5‑AS1 targeted several overlapping co‑expressed genes, including forkhead box F1 (FOXF1) and polycystin 1, transient receptor potential channel interacting (PKD1) in IDD [8]. In addition, the previous study have demonstrated that LncRNA OIP5-AS1 could inhibit osteoblast differentiation of valve interstitial cells via miR-137/TWIST11 axis, that OIP5-AS1 acted as a negative regulator of osteogenic differentiation [14]. Emerging data have revealed that LncRNA OIP5-AS1 were involved in the occurrence and development of osteoarthritis and downregulation of LncRNA OIP5-AS1 induced by IL-1βaggravates osteoarthritis via regulating miR-29b-3p/PGRN [15]. Nowadays, there is no study involving the part that OIP5-AS1 plays in IDD.

The interaction between microRNAs (miRNAs) and lncRNAs within cells has been shown to be one representative regulation pattern of miRNAs and should be discussed due to their large number. Studies have showed that the expression of lncRNAs can regulate the activities of miRNAs [16]. Here, miR-25-3p is investigated for its interaction with OIP5-AS1. MicroRNAs (miRs) are also another subclass of noncoding RNAs (ncRNA) below 25 nt in length [17,18]. Previous research studies have demonstrated that several miRNAs are dysregulated in IDD, including miR-21, miR-10b, miR-640, and miR-27 [19–22]. Reports have already revealed the promoting or inhibiting role of miR-25-3p in multiple diseases, including IDD. It has been delineated that miR-25-3p lowers expression of ADAM10 to hamper the formation of cardiomyocytes by P19 differentiation via inhibiting Notch signaling [23]. Besides, FOXD2-AS1 blocks miR-25-3p/Sema4C axis to boost the invasion and migration in colorectal cancer [24]. Previous researches have also demonstrated the suppressive role of miR-25-3p in tongue squamous cell carcinoma and closely associated with the degradation of human nucleus pulposus cells [25]. Moreover, miR-25-3p has been indicated to enhance NPCs proliferation and apoptosis resistance in IDD by reduction of Bim [26]. Therefore, it was speculated that OIP5-AS1 bound to miR-25-3p and accelerated the progression of IDD.

In this study, the expression pattern of OIP5-AS1 in IDD tissues was up-regulated. Function and downstream regulatory axis of OIP5-AS1 were further explored via CCK-8, EdU, flow cytometry, Western blot analyses. We observed that expression of OIP5-AS1 was markedly upregulated in IDD tissues and NPCs, and it exerted promoting effect through decreasing cell proliferation, as well as accelerating cell apoptosis, ECM degradation and inflammation response via targeting miR-25-3p.

## Materials and methods

### Tissue samples

The Ethics Committee of the Traditional Chinese Medical Hospital of Lianyungang approved this study in patients with IDD. All participants signed the written informed consent. The IVD tissues were collected as surgical waste from 65 patients with IDD. In addition, IVD tissues from patients with lumbar fractures, excluding those with spinal tumors, infections, and rheumatic immune diseases, were collected as control tissues. IDD tissues and control tissues were frozen in liquid nitrogen at −80°C. According to the MRI scans, IDD degree was classified utilizing the modified Pfirrmann classification (Table 1) [27]. The clinical samples characteristics were shown in Table 2.

**Table 1.** Pfirrmann grading standards in MRI of intervertebral disc degenerations

Grade	Structure	Border of nucleus pulposus and fibrous rings	Signal Intensity	Height of intervertebral disc
I	Homogeneous, bright white	Distinct	High signal or equal to CSF	Normal
II	Heterogeneous, with or without horizontal belt	Distinct	High signal or equal to CSF	Normal
III	Heterogeneous, gray	Ambiguous	Medium signal	Normal to moderately reduced
IV	Heterogeneous, gray or black	Disappear	Medium or low signal	Normal to moderately reduced
V	Heterogeneous, black	Disappear	Low signal	Disc collapse

**Table 2.** Samples characteristics

Characteristics	Value/n (%)
Age	48.3
Gender (female)	29 (44.6)
Gender (male)	36 (55.4)
Smoking	24 (36.9)
Disc degeneration grade	
II	10 (15.4)
III	13 (20)
IV	22 (33.8)
V	20 (30.8)

### Cell culture and treatment

Human degenerative NPCs used in this paper were extracted from NP tissues of IDD patients. The isolated NP tissues from three patients, which IDD degree is the same, were rinsed utilizing PBS three times and subsequently minced into pieces, followed by digestion via 0.25% trypsin solution for 30 min (Gibco, Carlsbad, CA, USA) and then 0.2% collagenase II (Crescent Chemical, Islandia, NY, USA) for 4 h at 37°C. Thereafter, NPCs were grown in DMEM/F12 (Gibco) with 10% FBS (Gibco), 100 U/mL penicillin as well as 100 mg/mL streptomycin under the condition of 37°C with 5% CO_2_. NPCs at the third passage were used for later experiments. 1 µg/ml of LPS (cat. no. L2630; Sigma-Aldrich) was utilized to stimulate NPCs in serum-free DMEM for 1 d, which could induce inflammation and matrix degradation in IVD.

### Cell transfection

Short harpin RNA targeting OIP5-AS1 (sh-OIP5-AS1; 5ʹ-CCGGGCTCCTAGGATTCCAGTTATCCTCGAGGCAGAAGGCTGAGTTTCATTTTTTTTG-3ʹ) with the negative control sh-NC (5ʹ-CACCGTTCTCCGAACGTGTCACGTCAAGAGATTACGTG ACACGTTCGGAGAATTTTTTG-3ʹ), miR-25-3p mimic (Sense 5ʹ-UCC CUG AGA CCC UAA CUU GUG A-3ʹ; antisense 5ʹ-ACA AGU UAG GGU CUC AGG GAU U-3ʹ) with mimic NC were obtained from GenePharma (Shanghai, China). Transfection in NPCs was conducted via Lipofectamine 2000 (Invitrogen) as per the instructions from the manufacturer.

### RT-PCR

Tissues or NPCs were prepared for isolation of total RNA using TRIzol reagent (Invitrogen) as per the manufacturer’s guidebook. The reverse transcription of 1-µg RNA into cDNA was carried out with the use of the 1st Strand cDNA Synthesis Kit (TAKARA). RT-PCR was conducted on the ABI 7500 real-time PCR system (Applied Biosystems) with the SYBR-Green PCR Master Mix (Roche) following the introductions. The parameters for PCR were: 10-min pre-treatment at 95°C; 40 cycles at 95°C for 15 sec; at 60°C for 1 min; and at 72°C for 5 min. The 2^−ΔΔCt^ method was applied for calculation of gene expression that was normalized to GAPDH or U6. Utilized primers were as follows: OIP5-AS1 sense, 5ʹ-GGTCGTGAAACACCGTCG-3ʹ and antisense, 5ʹ-GTGGGGCATCCAGGGT-3ʹ; Collagen IIsense, 5ʹ-CTGGTGATGATGGTGAAG-3ʹ and antisense, 5ʹ-CCTGGATAACCTCTGTGA-3ʹ; Aggrecan sense, 5ʹ-CAGATGGCACCCTCCGATAC-3ʹ and antisense, 5ʹ-GACACACCTCGGAAGCAGAA-3ʹ; GAPDH sense, 5ʹ-AATCCCATCACCATCTTCCAG-3ʹ and antisense, 5ʹ-TGATGACCCTTTTGGCTCCC-3ʹ; miR-25-3p sense, 5ʹ-CATTGCACTTGTCTCGGTCTGA-3ʹ and antisense 5ʹ-GCTGTCAACGATACGCTACGTAACG-3ʹ; U6 sense, 5ʹ-CTCGCTTCGGCAGCACA-3ʹ and antisense, 5ʹ-AACGCTTCACGAATTTGCGT-3ʹ.

### Immunofluorescence

NPCs were fixed utilizing 4% paraformaldehyde for just 0.5 h, cultured with 0.1% Triton X-100 for another 15 min, and subsequently blocked via applying 2% bovine serum albumin (Sigma) for extra 1 h. After that, NPCs were cultivated with primary antibody against collagen II (1:200; Santa Cruz Biotechnology) for whole night at 4°C, followed by exposure to secondary antibodies labeled with Alexa Fluor® 594 (1:100; Life Technologies) at indoor temperature for 1 h. After thrice washes by PBS, NPCs were stained via DAPI. Photos were observed by laser confocal microscopy (OLYMPUS).

### CCK-8 experiment

To assess cell viability, a commercial Cell Counting Kit-8 (CCK-8; Dojindo) was utilized. NPCs (5 × 10^3^ cells/well) were placed in 96-pore dishes and 10-µl CCK-8 solution was supplemented for extra culture of 2 h. Absorbance in each well was estimated at 450 nm.

### EdU assay

96-well plates (1 × 10^4^ cells/well) were prepared for culture of NPCs. For this assay, Cell-Light^TM^ EDU Apollo 488 kit (Ruibio, China) was employed. Cells were dyed with Hoechst 33,342. To assess cell proliferation rate, the ratio of the EdU positive cells/Hoechest staining cells selected randomly at three fields was calculated. Fluorescence microscope (Leica Microsystems) was utilized for observation.

### Flow cytometry

Apoptosis ability was evaluated via Annexin V-FITC-propidium iodide (PI) apoptosis detection reagent (BD Biosciences) as the manufacturer guided. Cells were first rinsed thrice utilizing PBS, digested using trypsin (1 ml) and next adjusted to the density of 1 × 10^5^ cells/100 µl for resuspension in 1X Annexin binding buffer. Next, cells were collected and dyed via applying the above reagent for 15 min. The apoptosis rate was quantified with flow cytometry 10.0 (FlowJo, FACS CaliburTM, BD Biosciences).

### Western blot

Total protein was extracted using a RIPA kit. Also, a BCA Protein Assay Kit (Thermo Scientific) was utilized for protein concentration. Equivalent protein (20 µg) was separated by employing 10% SDS-PAGE, which was transferred onto polyvinylidene fluoride (PVDF) membranes (Millipore). Membrane blockade was conducted using 5% BSA for 1 h at room temperature and incubated with primary antibodies against Collagen II, Aggrecan and GAPDH (1:1,000; Abcam) were adopted at 4°C for one night. Secondary culture was implemented with HRP-labeled goat anti-rabbit antibody (Boster, Wuhan, China). ECL Plus reagent (Millipore, USA) was adopted for visualization of signals. The intensity of bands was quantified via Image Lab 3.0 software (Bio-Rad, CA, USA). The experiment was repeated three times in each group.

### ELISA

The contents of IL-6 (cat: BMS213-2), TNF-α (cat: BMS223-4), IL-10 (cat: BMS215-2), and IL-1β (cat: BMS6002) were estimated through relative ELISA kits (eBioscience) following the producer’s instructions. Under the wavelength at 450 nm, all samples were assessed with a microplate reader (SpectraMax M5, Molecular Devices). Next, a standard curve was plotted with the help of computer software based on the absorbance value.

### Target gene analysis

LncRNA target gene predictions were made through the use of the StarBase (http://starbase.sysu.edu.cn/index.), miRDB (http://mirdb.org/), TargetScan (http://www.targetscan.org) and miRWalk (http://mirwalk.umm.uni-heidelberg.de/) lncRNA-target gene prediction databases.

### Luciferase reporter assay

The wild- and mutant-type OIP5-AS1 with or without putative-binding sites for miR-25-3p (OIP5-AS1-WT/MUT) were constructed into pmiRGLO (Invitrogen). After that, NPCs were co-transfected with miR-25-3p or miR-NC with OIP5-AS1-WT or OIP5-AS1-MUT, the procedures of which were implemented utilizing Lipofectamine 2000 (Invitrogen). After 48 h post transfection, the luciferase activity in each group was analyzed using a dual-luciferase reporter system (Promega). Renilla luciferase activity acted as the normalization.

### Statistical analysis

The gained data of triplicated experiments were analyzed by SPSS version18.0 software (SPSS Inc., Chicago, IL, USA) and represented as the mean ± standard deviation (SD). Comparisons between or among groups were analyzed via Student’s t-test or one-way ANOVA. The statistics were significant upon P < 0.05.

## Results

### OIP5-AS1 was up-regulated in IDD

**Figure 1.**
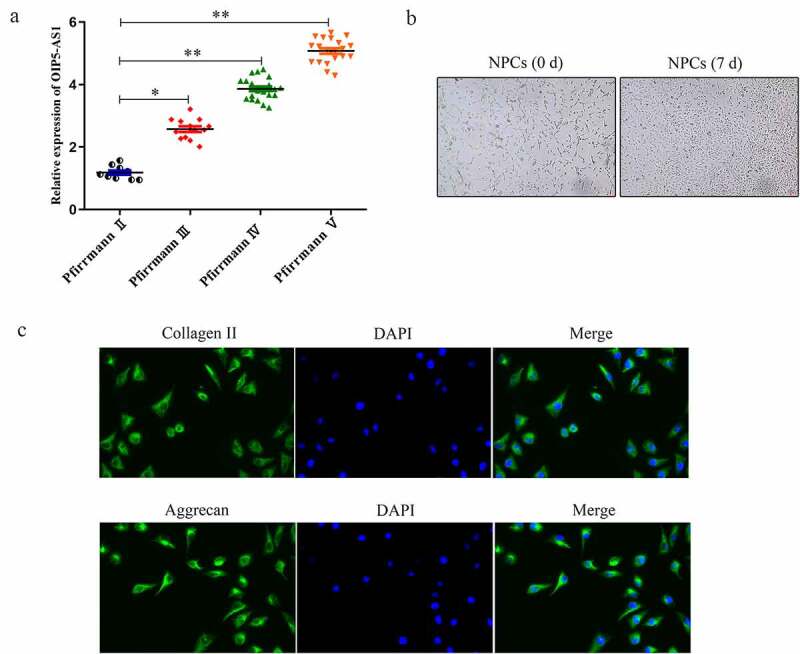
**OIP5-AS1 was up-regulated in IDD and its silence**. (a) RT-PCR analysis of OIP5-AS1 in groups of different Pfirrmann scores. *P < 0.05, **P < 0.01 *vs*. Pfirrmann II. (b) Cell morphology of NPCs isolated from IDD tissues. (c) Immunofluorescence for Collagen II and Aggrecan expression in NPCs was conducted

Firstly, expression of lncRNA OIP5-AS1 in tissues of IDD patients was assessed. RT-PCR delineated that the positive correlations between OIP5-AS1 levels and the patients’ Pfirrmann scores from II to V was affirmed, hinting that high expression of OIP5-AS1 suggested more severe degree of IDD (Figure 1(a)). As presented in Figure 1(b), NPCs isolated from tissues were star-shaped, polygonal or spindle-shaped, similar to chondroid cells. Besides, immunofluorescence assay illustrated that Collagen II and Aggrecan expression were expressed at low level in NPCs (Figure 1(c)).

### OIP5-AS1 silence accelerated cell proliferation and decreased cell apoptosis and ECM degradation

**Figure 2.**
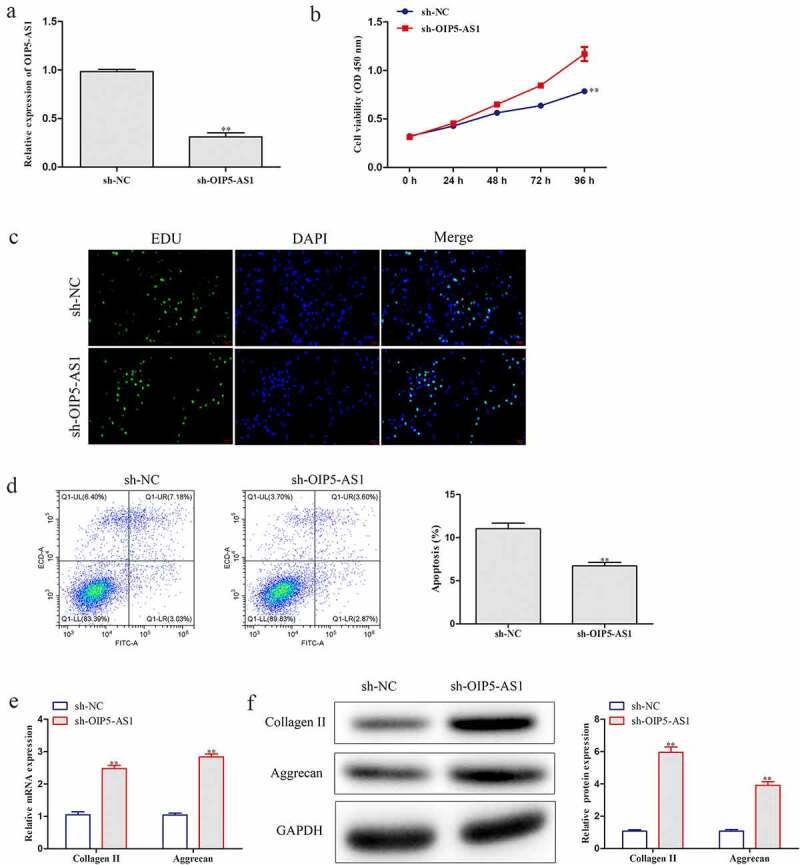
**OIP5-AS1 silence accelerated cell proliferation and decreased cell apoptosis and ECM degradation**. (a) Level of OIP5-AS1 in NPCs was estimated via RT-PCR experiment. **P < 0.01 *vs*. sh-NC. (b-c) CCK-8 and EdU experiments for analyzing cell proliferation ability of NPCs treated with sh-NC or sh-OIP5-AS1. (d) The apoptotic levels of NPCs after OIP5-AS1 silence were explored through flow cytometry. (e-f) RT-PCR and Western blot were conducted to assess mRNA and protein expression of Collagen II and Aggrecan in OIP5-AS1-silenced NPCs. **P < 0.01 *vs*. sh-NC

We further explored the function of OIP5-AS1 in IDD. RT-qPCR was utilized to analyze the level of OIP5-AS1 in NPCs transfected with sh-OIP5-AS1. OIP5-AS1 was dramatically lowered after sh-OIP5-AS1 treatment (Figure 2(a)). CCK-8 experiment proved that cell viability of NPCs was distinctly promoted by OIP5-AS1 silence (Figure 2(b)). Also, boosted cell proliferation by OIP5-AS1 knockdown was observed in NPCs through EdU assay (Figure 2(c)). It was clear that OIP5-AS1 was heightened in IDD and silence of OIP5-AS1 promoted cell proliferation.

hen, the effect of OIP5-AS1 on cell apoptosis was assessed. Flow cytometry analysis revealed that inhibition of OIP5-AS1 remarkably hampered cell apoptosis (Figure 2(d)). Besides, RT-PCR and Western blotting were performed to investigate the changes of Collagen II and Aggrecan, which presented that mRNA and protein expression of Collagen II and Aggrecan was obviously increased under OIP5-AS1 silencing compared with si-NC group (Figure 2(e-f)). Here, cell apoptosis and ECM degradation were inhibited when OIP5-AS1 was silenced.

### Decrease of OIP5-AS1 reduced inflammation response in LPS-induced NPCs

**Figure 3.**
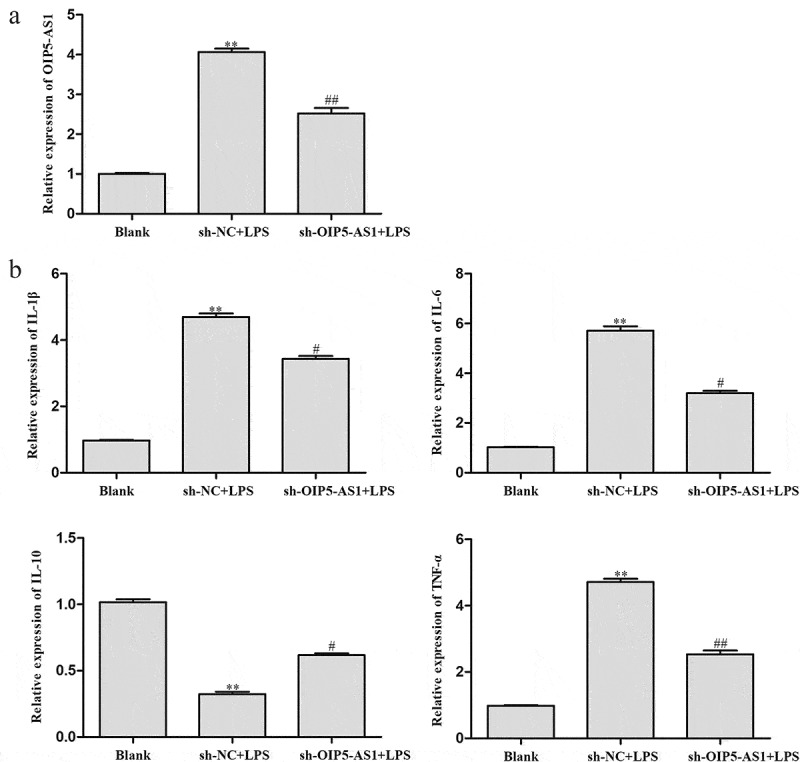
**Decrease of OIP5-AS1 declined inflammation response in LPS-induced NPCs**. (a) Levels of OIP5-AS1 in NPCs of Blank, sh-NC+LPS and sh-OIP5-AS1+ LPS groups were detected through RT-PCR. (b) Levels of IL-6, TNF-α, IL-10 and IL-1β were explored via ELISA. **P < 0.01 *vs*. Blank, ^#^*P* < 0.05, ^##^*P* < 0.01 *vs*. sh-NC+LPS

Next, we investigated the role of OIP5-AS1 in LPS-induced NPCs. RT-PCR demonstrated that LPS induced the expression of OIP5-AS1, and OIP5-AS1 expression was further down-regulated by transfection of sh-OIP5-AS1 (Figure 3(a)). As measured by ELISA, the levels of IL-6, TNF-α, and IL-1β were significantly elevated but the level of IL-10 was significantly lowered after LPS stimulation. However, OIP5-AS1 knockdown markedly reversed the up-regulation of IL-6, TNF-α and IL-1β levels and the depletion of IL-10 level (Figure 3(b)). Obviously, the inflammation was suppressed with OIP5-AS1 down-regulation.

### MiR-25-3p was targeted by OIP5-AS1 and low in IDD

**Figure 4.**
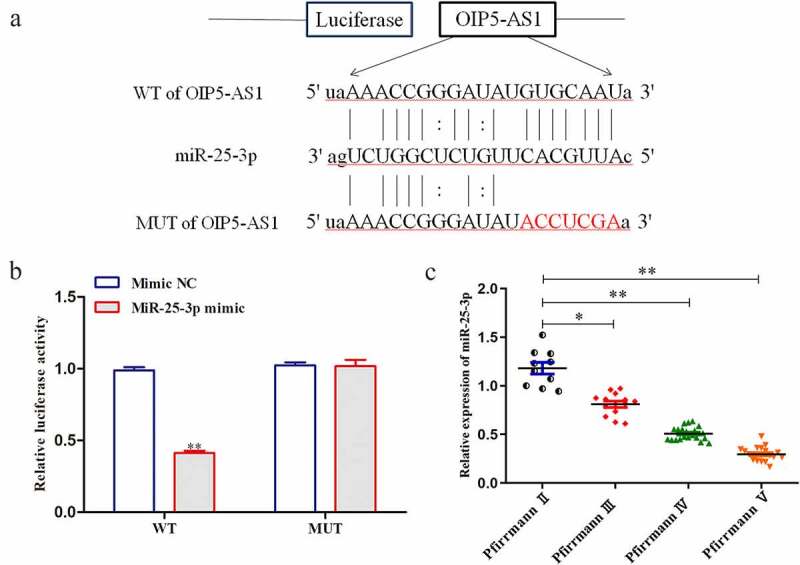
**MiR-25-3p was targeted by OIP5-AS1 and it was low in IDD**. (a) Binding sites between OIP5-AS1 and miR-25-3p as predicted by bioinformatic tools. (b) Luciferase reporter assay was applied to determine the interaction of OIP5-AS1 with miR-25-3p. **P < 0.01 *vs*. Mimic NC. (c) RT-PCR was employed to evaluate the level of miR-25-3p in tissues with various Pfirrmann scores. **P < 0.01 *vs*. PfirrmannII

The downstream target genes of OIP5-AS1 were predicted using starbase (http://starbase.sysu.edu.cn/). It was predicted that OIP5-AS1 bound with miR-25-3p. The binding sites were shown in Figure 4(a). In luciferase reporter assay, it was validated that the luciferase activity of OIP5-AS1-WT was overtly decreased when miR-25-3p was overexpressed. In contrast, that of OIP5-AS1-MUT had no changes in response to miR-25-3p elevation (Figure 4(b)). The expression of miR-25-3p negatively correlated with Pfirrmann scores (Figure 4(c)). Taken together, miR-25-3p was lowly expressed in IDD and was target of OIP5-AS1.

### Reduction of miR-25-3p rescued the effects of OIP5-AS1 inhibition on proliferation, apoptosis, and ECM degradation

**Figure 5.**
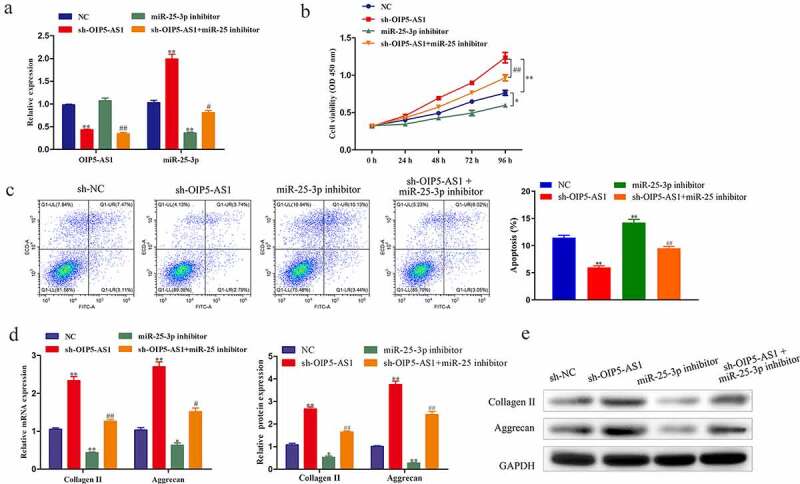
**Reduction of miR-25-3p rescued the effects of OIP5-AS1 inhibition on proliferation, apoptosis and ECM degradation**. (a) Levels of OIP5-AS1 and miR-25-3p under different transfections were explored through RT-PCR. (b) CCK-8 detection of cell proliferation of NPCs. (c) Flow cytometry test of apoptotic levels in NPCs. (d) RT-PCR and Western blotting for analyzing Collagen II and Aggrecan expression in mRNA and protein levels in NPCs. *P < 0.05, **P < 0.01 *vs*. NC, ^#^*P* < 0.05 and ^##^P < 0.01 *vs*. sh-OIP5-AS1

To probe whether OIP5-AS1/miR-25-3p axis functioned in IDD, we conducted rescue experiments. As affirmed through RT-PCR, miR-25-3p silence had no effect on OIP5-AS1 expression. Whereas, OIP5-AS1 downregulation significantly promoted miR-25-3p expression, which was distinctly reduced with treatment of miR-25-3p inhibitor (Figure 5(a)). CCK-8 results indicated that decrease of miR-25-3p distinctly repressed cell viability. Moreover, the accelerated cell viability due to OIP5-AS1 depletion was hindered by miR-25-3p inhibition (Figure 5(b)). Flow cytometry analysis elucidated that cell apoptosis was markedly repressed by OIP5-AS1 knockdown, and the obstruction was abolished with miR-25-3p inference (Figure 5(c)). The mRNA and protein levels of Collagen II and Aggrecan was decreased under miR-25-3p suppression but promoted under OIP5-AS1 inference, which was neutralized with co-treatments of sh-OIP5-AS1 and miR-25-3p inhibitor (Figure 5(d-e)). In summary, the promotion of proliferation and the inhibition of apoptosis and ECM degradation caused by reduction of OIP5-AS1 were abrogated with silencing of miR-25-3p.

### Reduction of miR-25-3p rescued the effects of OIP5-AS1 inhibition on the inflammation

**Figure 6.**
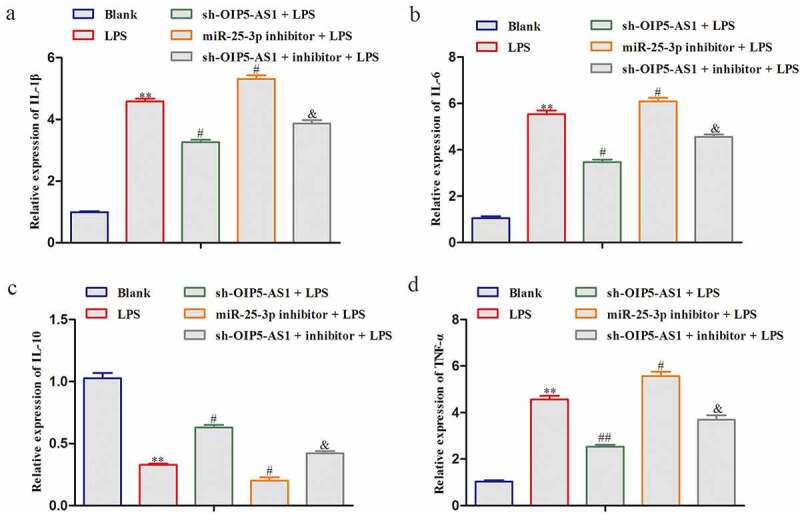
**Reduction of miR-25-3p rescued the effects of OIP5-AS1 inhibition on the inflammation**. ELISA for contents of IL-6, TNF-α, IL-10 and IL-1β in LPS-induced NPCs. **P < 0.01 *vs*. Blank, ^#^*P* < 0.05 and ^##^*P* < 0.01 *vs*. LPS, ^&^*P* < 0.05 *vs*. LPS+sh-OIP5-AS1

In LPS-treated NPCs, the regulatory mechanism of OIP5-AS1/miR-25-3p axis was explored. Reduction of miR-25-3p improved the levels of IL-6, TNF-α, and IL-1β whereas reduced the IL-10 level in LPS-treated NPCs. And the decreases of IL-6, TNF-α, and IL-1β and the increase of IL-10 attributed to inference of OIP5-AS1 were significantly recovered when miR-25-3p was lessened (Figure 6). Collectively, the inhibition of inflammation response caused by reduction of OIP5-AS1 was abrogated with silencing of miR-25-3p.

## Discussion

Through searching on NCBI website, we discovered that lncRNA OIP5-AS1 is a promotive molecule in different diseases, and these abnormally expressed LncRNAs can be used as biomarkers for early clinical diagnosis. During the process of atherosclerosis, lncRNA OIP5-AS1 targets miR-26a-5p to modulate AKT/NF-κB pathway and exerts promotive effect [28]. LncRNA OIP5-AS1 indicates dismal prognosis and mediates cell proliferation in bladder cancer through OIP5 [29]. Additionally, OIP5-AS1 decrement boosts cell viability, suppresses apoptosis, and inhibits LDH release in HUVECs treated by ox-LDL [13]. However, its expression pattern and biological role in IDD have not been well studied. In the present study, we firstly explored the level of OIP5-AS1 in IDD tissues. Compared with control tissues, experimental data unmasked that OIP5-AS1 was high expressed in IDD tissues. Furthermore, OIP5-AS1 level presented increased trend with the development of IDD. Thus, it was reasonable to suppose that OIP5-AS1 played a vital role in IDD, and it was chosen for further investigation in IDD.

The etiology of IDD composes of the reduction of NPCs caused by pathological changes such as apoptosis activation, which leads to ECM degradation and degeneration [30,31]. The unique structural hallmark of IDD is responsible for its ability in load-bearing and the correct mechanical stress transmission to the adjacent vertebrae. The major processes involved in ECM degeneration are the dysregulation in the synthesis of fundamental matrix components by IDD cells, as well as the increased production of degradative enzymes. Proteoglycans (primarily aggrecan) and Collagen II are two main effectors of ECM, the decreases of which can promote the degradation of ECM [32]. IDD is characterized by the early onset of a severe inflammatory milieu both inside the degenerating IVD and in the peridiscal space. A relevant number of studies have been conducted on disc samples with different degrees of degeneration and revealed the levels of several inflammatory cytokines and chemokines was increased during IDD [33]. In the current paper, it was affirmed that under OIP5-AS1 depletion, promoted cell proliferation, inactivated cell apoptosis and inhibited ECM degradation were observed. Besides, increasing expression of inflammatory cytokines also contributes to IDD degeneration [34,35]. The impacts of OIP5-AS1 on inflammatory factors were investigated. In LPS-treated NPCs, the elevated levels of inflammatory factors were markedly decreased with the inference of OIP5-AS1. The functional role of OIP5-AS1 in IDD was firstly unveiled by the current work. Taken together, OIP5-AS1 inference played a suppressive role in IDD. To further verify the mechanism of OIP5-AS1 in IDD, functional experiments were carried out. Loss-of-function experiments suggested that OIP5-AS1 played a vital role in IDD. On the one hand, after OIP5-AS1 expression was knocked down, the cell proliferation was enhanced. On the other hand, knockdown of OIP5-AS1 could impede the apoptosis as well as ECM degradation, and reduced the inflammation response.

Multiple studies have demonstrated the mechanism of lncRNAs with miRNAs in IDD. For examples, lncRNA HCG18 potentiates IDD via sponge of miR-146a-5p and regulation of TRAF6 [36]. LINC00641 modulates autophagy and IDD by interaction with miR-153-3p under stress from nutrition deprivation [37]. HOTAIR acts as a miR-34a-5p sponge to inactivate apoptosis of NPCs via NOTCH1 signaling [38]. In this research, we utilized bioinformatic tool to predict the potential genes interplaying with OIP5-AS1, we found that OIP5-AS1 sponged miR-25-3p. Interestingly, miR-25-3p, assumed to be targeted by OIP5-AS1, has been reported as a suppressor for IDD [26]. Additionally, although the expression of OIP5-AS1 and miR-25-3p has been testified, such relationship was first identified in IDD until our study.

In this study, we also observed that the expression level of miR-25-3p in IDD tissues was also dramatically down-regulated. Also, with the increasing degree of IDD, level of miR-25-3p gradually reduced. Next, mechanism experiments were conducted. Luciferase reporter assays verified the binding of OIP5-AS1 to miR-25-3p in IDD. Additionally, elevated expression of miR-25-3p was observed upon repression of OIP5-AS1, hinting the inhibitory effect of OIP5-AS1 on miR-25-3p. Further, the function of OIP5-AS1/miR-25-3p was explored via reducing the expression of miR-25-3p in the presence of OIP5-AS1 inhibition. The results demonstrated that depletion of miR-25-3p neutralized the effects of OIP5-AS1 decrease on proliferation, apoptosis and ECM degradation. Moreover, miR-25-3p reduction rescued the effects of OIP5-AS1 decrement on inflammation cytokines. These findings affirmed that OIP5-AS1 repressed miR-25-3p expression to suppress proliferation, as well as facilitate apoptosis, ECM degradation and inflammation in IDD.

## Conclusions

In summary, we indicated the high expression of OIP5-AS1 in IDD, and disclosed the simulative impact of OIP5-AS1 on proliferation and its suppressive impacts on apoptosis, ECM degradation, and inflammation. MiR-25-3p was verified to be the target of OIP5-AS1 and its depletion could reverse the effects of OIP5-AS1 on the above pathological changes. The present work provides evidence that OIP5‑AS1 targets miR-25-3p to facilitate IDD, which is possibly beneficial for IDD therapy. I will further investigate the pathogenesis of IDD in relation to OIP5‑AS1 and its underlying mechanisms using series of experiments with NPC samples and in vivo model in the future.

## Data Availability

The datasets used and/or analyzed during the present study are available from the corresponding author on reasonable request.
